# Effectiveness of a psychological intervention delivered by general nurses for alcohol use disorders in people living with HIV in Zimbabwe: a cluster randomized controlled trial

**DOI:** 10.1002/jia2.25641

**Published:** 2020-12-13

**Authors:** Munyaradzi Madhombiro, Martin Kidd, Bazondlile Dube, Michelle Dube, Wilson Mutsvuke, Thabani Muronzie, Danai Tavonga Zhou, Sarah Derveeuw, Dixon Chibanda, Alfred Chingono, Simbarashe Rusakaniko, Alan Hutson, Gene D Morse, Melanie A Abas, Soraya Seedat

**Affiliations:** ^1^ Department of Psychiatry University of Zimbabwe Harare Zimbabwe; ^2^ SUNY University at Buffalo Buffalo NY USA; ^3^ Centre for Statistical Consultation Stellenbosch University Stellenbosch South Africa; ^4^ Department of Medical Laboratory Sciences University of Zimbabwe Harare Zimbabwe; ^5^ Institute of Psychiatry, Psychology and Neuroscience King’s College London London United Kingdom; ^6^ Department of Biostatistics and Bioinformatics Roswell Park Comprehensive Cancer Center Buffalo NY USA; ^7^ SUNY University at Buffalo Buffalo NY USA; ^8^ Lewisham Hospital South London and Maudsley NHS Foundation Trust London United Kingdom; ^9^ Faculty of Medicine and Health Sciences Stellenbosch University Stellenbosch South Africa

**Keywords:** LMIC, risk factors, Africa, alcohol disorders, cognitive behavioural therapies, motivational interviewing

## Abstract

**Introduction:**

There have been very few randomized clinical trials of interventions for alcohol use disorders (AUD) in people living with HIV (PLWH) in African countries. This is despite the fact that alcohol use is one of the modifiable risk factors for poor virological control in PLWH on antiretroviral therapy.

**Methods:**

Sixteen clinic clusters in Zimbabwe were selected through stratified randomization and randomized 1: 1 to Intervention and Control arms. Inclusion criteria for individual participants were being adult, living with HIV and a probable alcohol use disorder as defined by a score of 6 (women) or 7 (men) on the Alcohol Use Disorders Identification Test (AUDIT). In the Intervention clusters, participants received 8 to 10 sessions of Motivational Interviewing blended with brief Cognitive Behavioural Therapy (MI‐CBT). In the control clusters, participants received four Enhanced Usual Care (EUC) sessions based on the alcohol treatment module from the World Health Organisation mhGAP intervention guide. General Nurses from the clinics were trained to deliver both treatments. The primary outcome was a change in AUDIT score at six‐month post‐randomization. Viral load, functioning and quality of life were secondary outcomes. A random‐effects analysis‐of‐covariance model was used to account for the cluster design.

**Results:**

Two hundred and thirty‐four participants (n = 108 intervention and n = 126 control) were enrolled across 16 clinics. Participants were recruited from November 2016 to November 2017 and followed through to May 2018. Their mean age was 43.3 years (SD = 9.1) and 78.6% (n = 184) were male. At six months, the mean AUDIT score fell by −6.15 (95% CI −6.32; −6.00) in the MI‐CBT arm, compared to a fall of − 3.09 95 % CI − 3.21; −2.93) in the EUC arm (mean difference −3.09 (95% CI −4.53 to −1.23) (*p* = 0.05). Viral load reduced and quality of life and functioning improved in both arms but the difference between arms was non‐significant.

**Conclusions:**

Interventions for hazardous drinking and AUD comprising brief, multiple alcohol treatment sessions delivered by nurses in public HIV facilities in low‐income African countries can reduce problematic drinking among PLWH. Such interventions should be integrated into the primary care management of AUD and HIV and delivered by non‐specialist providers. Research is needed on cost‐effectiveness and implementation of such interventions, and on validation of cut‐points for alcohol use scales in low resource settings, in partnership with those with lived experience of HIV and AUD.

## 1 INTRODUCTION

For people living with HIV, Alcohol Use Disorders (AUD) are highly prevalent and increase the likelihood of risky sexual practices, poor engagement in HIV care, viral non‐suppression and liver damage [1, 2, 3, 4]. Out of the 34 million people living with HIV (PLWH) worldwide, 68% live in sub‐Saharan Africa (SSA) [5]. Among PLWH in SSA, a recent meta‐analysis estimated the prevalence of AUD to range between 12% and 62% [6]. Binge drinking is more frequent in PLWH living in SSA than in the general population [7]. All these findings point to the need for research on interventions to reduce alcohol use in PLWH living in SSA.

AUD treatment involves screening and provision of psychosocial interventions, with or without pharmacological therapies. In high‐income countries, several psychological interventions are used for AUD including Motivational Interviewing (MI) alone or with cognitive behavioural therapy (CBT), stress management, problem solving, case management and community contingency therapy [8, 9, 10]. Until recently, however, there has been little evidence that any of these interventions have beneficial effects on AUD in PLWH. MI, plus personalized feedback on the level of alcohol intake and its potential harm to the individual showed promise in two studies in the United States [8, 9]. Given the shortage of psychologists in low‐ and middle‐income countries (LMICs), the World Health Organisation (WHO) developed the Mental Health Gap Intervention Guide (mhGAP IG) [11]. All the interventions in the WHO mhGAP IG are evidence‐based and can be delivered by non‐specialists. The WHO mhGAP IG recommends brief (one‐session) psychoeducation for AUD in general primary care. However, there is a dearth of research on mhGAP interventions for PLWH in SSA.

Until 2020 there had only been five published randomized controlled trials (RCT) of interventions for AUD in PLWH in SSA [10, 12, 13, 14, 15] all with limitations. One evaluated a four‐session intervention in a small sample of women [10]. Another compared six sessions of CBT with usual care but the sample size was small (n = 75 and the follow‐up period relatively short (90 days) [13]. Another found a 20‐session intervention reduced alcohol‐use compared to a waitlist control [15] but this model is unlikely to be sustainable. Two studies found a single‐session intervention to be no better than usual care [12, 14]. Since these, Papas et al (2020) [16], in a large RCT (n = 614) in Kenya (n = 614), found a culturally adapted 6‐session group CBT intervention delivered by paraprofessional counsellors to be superior to a Healthy Lifestyles education intervention in reducing percent drinking days and mean drinks per day at nine‐month follow‐up. Zimbabwe has a disproportionately high burden of HIV of 1.3 million PLWH in a population of 16 million and over six litres of alcohol consumption per capita, yet no systematic screening of alcohol use in PLWH is routinely undertaken at HIV care clinics [17, 18]. Interventions for alcohol use in Zimbabwe should include screening for regular and recent alcohol consumption as a risk factor for HIV acquisition in HIV counselling and testing settings. Screening and counselling for alcohol use have the potential to improve HIV treatment outcomes in PLWH who have an AUD in these settings.

We have previously described the adaptation and preliminary testing of an intervention based on MI and simple CBT for AUD in an HIV care clinic in Zimbabwe [19, 20]. Formative qualitative work identified stigma and the time commitment required by patients as being the main impediments to uptake [21]. Furthermore, our pilot and feasibility RCT provided preliminary evidence that interventions targeting AUD could be effectively delivered by nurses; could reduce alcohol use and improve immunological parameters in PLWH [22]. In studies of PLWH, quality of life and functioning have been shown to be both predictors and outcomes of virological suppression and should be assessed [23, 24, 25]. Our aim here was to compare an adapted nurse‐delivered intervention based on MI and CBT techniques (MI‐CBT) with nurse‐delivered brief psychoeducation based on the mhGAP IG. We hypothesized that MI‐CBT would be significantly superior to EUC in improving both primary and secondary outcomes. We aimed to assess changes in AUD, viral load, functioning and quality of life.

## 2 METHODS

### 2.1 Setting and sampling

In Zimbabwe, HIV care has been decentralized outside tertiary hospitals to increase access to antiretroviral therapy. Nationally, there are 109 medium‐sized facilities comprising 7 provincial, 47 district and 25 church‐related hospitals and 30 large urban primary care polyclinics. In order to generate a representative sample, clusters were stratified by the number of patients registered for HIV care. Based on these stratification criteria, 16 facilities were randomly selected for the trial: two provincial hospitals, six district hospitals, five church‐related hospitals and three polyclinics. We used a computer‐generated randomization schedule to allocate clusters to MI‐CBT and EUC arms in a 1:1 ratio.

### 2.2 Sample size

The sample size calculation was based on a meta‐analysis of brief interventions for alcohol use disorders [26] on literature guiding the estimation of sample size for cluster RCT [27] and on data obtained from our pilot study [19]. The total sample size of 16 clusters, 8 clusters per arm, each with 15 participants enrolled per cluster giving a total sample of 240 (120 per arm), provided 80% power to detect a mean score difference of 2.5 on the AUDIT (with precision of ±0.45) between the treatment conditions (with a standard deviation within a cluster of 4), assuming an intra‐cluster correlation of 0.02 and taking into account a design effect of 1.56 and an attrition rate of 30% Blinding. Registered general nurses who provided the treatments and the principal investigators were not blinded to the treatment arms. However, the graduate‐level research assistants who recruited participants and performed baseline and outcome measures were blinded to the treatment arms.

### 2.3 Participants

PLWH with an AUD, on combination antiretroviral therapy, were recruited at HIV clinics. Inclusion criteria were as follows: at least 18 years of age; on antiretroviral therapy for at least three months; score of ≥6 (for women) or ≥7 (for men) on the Alcohol Use Identification Test (AUDIT) and free of cognitive impairment as assessed with the International HIV Dementia Scale (score more than 10).

### 2.4 Recruitment

We developed a computerized data of all adults attending HIV care at the 16 facilities. For each facility, 450 adults were randomly selected to be contacted for possible participation. Trained recruiters contacted individuals at the facilities by phone or in person with assistance from outreach services at the facilities. Those with any reported alcohol use were invited to screen for the study using the AUDIT. Those with AUDIT scores above the cut point and meeting all eligibility criteria were enrolled. The target was 15 participants per facility.

### 2.5 Intervention

Participants received MI‐CBT, which has been previously piloted in Zimbabwe [19]. Based on our past feasibility study, we split the original four sessions into two to make the content more deliverable for nurses within their usual consultation sessions, thus extending the intervention to eight sessions. The components of MI‐CBT were derived from Project MATCH Motivational Enhancement Therapy (MET) and Cognitive Behavioural Therapy (CBT) manuals [28, 29]. The intervention comprised up to 10 sessions with each session lasting 45 to 60 minutes. Session 1 included personalized feedback on the participants’ AUDIT score with education about the interpretation of different AUDIT cut‐off scores. Personalized feedback is simple and limited to information giving – it involves telling the person what their AUDIT score is and then explaining what that signifies, based on the WHO AUDIT guide. Session 1 also included feedback on the viral load, explaining the implications of a high viral load. Education was then provided about the link between AUDIT score and viral load. The nurse worked with participants to set their own alcohol reduction goals and their own HIV treatment goals. Session 2 included exploration of participants’ reasons for alcohol use, and assessment of their current stage of change. In session 2, the nurse reinforced the link between alcohol use and viral load. Session 2 included a review of participants’ alcohol reduction goals and their HIV treatment goals. In most cases, the goal was reduction rather than abstinence. Finally, in Session 2, participants’ life goals were elicited. Session 3 included a review of participants’ alcohol reduction goals, their HIV treatment goals and their life goals. Session 3 also included a discussion of the pros and cons of changing alcohol use. Session 4 included a review of participants’ alcohol reduction goals, their HIV treatment goals and their life goals, and included brainstorming around the difficulties in moving towards each of the goals. As participants moved towards change, Session 4 further included advice about dealing with situations where they would be at risk of excessive consumption. In the Zimbabwean context; this included such techniques as drinking alcohol slowly, being the last person to finish their alcoholic drink, drinking water instead of alcohol, eating food before consuming any alcohol and alternating alcoholic drinks with drinking water. Session 5 included a review of participants’ alcohol reduction goals, their HIV treatment goals and their life goals, and brainstorming around difficulties in moving towards each of the goals. Session 5 also included a discussion of situations in which drinking was unavoidable and also sought to identify triggers for relapse. Session 6 included a discussion on dealing with the challenges around HIV treatment, such as running out of antiretroviral therapy, HIV complications and coping with life. Sessions 7 and 8 included planning for the future, anticipating challenges, and discussing further treatment for HIV and/or alcohol‐related difficulties, as needed. For each session attended, participants were reimbursed for their bus fare and given $3 compensation in line with local IRB standards. We added two sessions for personalized feedback about personal goals on alcohol use and HIV treatment outcomes at three and six months, bringing the total maximum number of sessions to ten.

### 2.6 Control

Participants received enhanced usual care group (EUC) comprising care based on the alcohol‐use module of the WHO Mental Health Gap Intervention guide (WHO mhGAP IG) [30]. The WHO mhGAP IG has previously been used in other studies as an active control [11]. The EUC consisted of individual feedback on participants’ AUDIT score, their viral load and CD4 count results and psychoeducation on safe drinking. This lasted two to three hours, divided into one or two sessions. We added two sessions to provide personalized feedback on alcohol use and HIV treatment effectiveness at three and six months.

### 2.7 Supervision

Registered general nurses received supervision from BD who is a trained master’s level mental health nurse. Supervision was both provided in person and over the phone. The sessions lasted between 45 to 70 minutes depending on the individual intervention staff needs. The supervision sessions occurred between session 1 and 4, then at month 3 and 6. Specific components of supervision included assessment of compliance with the intervention protocol as well as administrative compliance (i.e. matching of participant records with the intervention nurse records, duration of sessions as obtained on the audio‐tapes, matching participant sign‐offs for reimbursements with receipts and staff records). Where there were protocol or administrative violations, corrective measures were taken.

### 2.8 Outcomes

The primary outcome was a change in AUDIT score from baseline to six months. Secondary outcomes were the change in viral load, CD4 count, functionality, as measured by the World Health Organisation Disability Schedule 2.0 (WHODAS 2.0) score and quality of life as measured by the World Health Organisation Quality of Life HIV (WHOQoL HIV) score from baseline to six months.

### 2.9 Measures

#### 2.9.1 Alcohol use

The Alcohol Use Disorders Identification Test (AUDIT) is a ten‐question scale which can be used as an interview or as a self‐report tool. It was developed by the WHO to screen patients for possible unhealthy alcohol consumption for use in primary care settings [31, 32].The AUDIT has three questions on alcohol consumption, three questions on drinking behaviour and dependence and four questions on the consequences or problems related to drinking [33, 34, 35]. Although the AUDIT has not been validated in Zimbabwe, several studies have utilized the AUDIT as an instrument to assess alcohol use in various communities including Zimbabwe [36, 37, 38]. A recent systematic review found a variety of cut‐off points being used [39]. The AUDIT has been found to perform well in detecting hazardous drinking at scores from >3 to >5; for harmful drinking from >5 to >16; and for dependent drinking from >7 to >24 [40], with differential cut‐off points for females and males [41]. In this study we used a cut‐off point of 6 for females and 7 for males, based on studies of harmful drinking and hazardous drinking [39]. We chose this slightly lower cut‐off point, informed by the literature, on the grounds that the negative effects of alcohol on physiologic damage are worse in people living with HIV [6] than those not infected and because our pilot study suggested that locally brewed drinks are high in alcohol content in Zimbabwe [22]. Countries vary as to the unit or amount of alcohol in a standard drink [42], but this information is not yet available for Zimbabwe.

#### 2.9.2 Adherence to HIV treatment

Adherence to HIV treatment was measured as a percentage of scheduled visits for collection of medication refills in the past three months which was collated from routine pharmacy records [44]

#### 2.9.3 Viral Load and CD4

Viral load (copies/mL) and CD4 count (absolute number of cells per cubic liter) were measured from whole blood at baseline and at six months (i.e. at the completion of the follow‐up period). Absolute values for viral load were log‐transformed for the analysis due to skewness of the data. A suppressed viral load was defined as <40 copies/mL on the COBAS AmpliPrep^®^ platform. All samples were analyzed by the University Of Zimbabwe Department Of Medicine Infectious Disease Laboratory.

#### 2.9.4 Disability

The World Health Organisation Disability Assessment Schedule‐2.0 (WHODAS 2.0) was used to assess for functional disability. This is a short, face‐valid measure of self‐reported disability, developed to measure disease burden across all psychiatric and medical diseases, across populations and cultures [43]. The WHODAS 2.0 incorporates 6 domains: cognition, mobility, self‐care, getting along, life activities and participation [43]. Although the WHODAS 2.0 has not been validated in Zimbabwe, it has been used with PLWH HIV patients in SSA [43].

#### 2.9.5 Quality of life

The WHO Quality of Life in HIV (WHOQoL HIV) was used to assess quality of life [44]. This tool has six domains that include physical, psychological, level of independence, social relationships, environment and spirituality domains. Although the WHOQoL has not been validated in Zimbabwe, the tool has been used in the region [44].

#### 2.9.6 Procedure

Registered general nurses (RGNs) were trained, with one team providing training in MI‐CBT and another team providing training in the EUC. The training included PowerPoint presentations, quizzes and role‐playing. Training took place at the clinics which allowed the RGNs to integrate the training into their usual schedule. Training in the MI‐CBT took a full day of seven hours, while training in the EUC took three hours. Each participant was allocated to a specific nurse who delivered the full course of treatment. Appointments were scheduled to avoid disruption of normal clinic activities. RGNs were compensated for their time (at US $5 per session) to deliver the therapies.

#### 2.9.7 Fidelity

Treatment sessions were recorded by hand, and approximately 10% of the sessions were audio‐recorded after consent was given, at least one session per client. Audio‐recorded sessions were used to provide feedback to the nurses to improve their competencies in the interventions and to help maintain the fidelity to treatment delivery. The two study teams visited each clinic for two separate days during the first three months of the study to provided supervision sessions.

#### 2.9.8 Ethics

All procedures involving human subjects/patients were approved by Stellenbosch Health Research Ethics Committee (HREC) and the Medical Research Council of Zimbabwe (MRCZ) ‐ approval: (SI/10/14/222) and (A/1936). The clinical trial registration number is PACTR201509001211149 – registered with https://pactr.samrc.ac.za/.

#### 2.9.9 Statistical analysis

All participants were included in the analysis of primary and secondary outcomes. For baseline assessments, descriptive statistics including means and their standard deviations, medians and their interquartile ranges were used. Chi^2^ were used for categorical and t‐tests were used for numerical variables. To account for the stratified cluster trial design, a random‐effects analysis‐of‐covariance model was utilized with fixed effects for baseline value and treatment arm, a random effect for treatment arm nested in clinic and a random intercept relative to the regression component of the ANCOVA model of the six‐month level as a function of the baseline value within cluster. Independent t‐tests were used to compare the baseline characteristics of the two groups. Analyses were undertaken using SAS 9.4 [45]. All tests were two‐sided with an alpha = 0.05. All outcome measures (AUDIT score, WHODAS, WHOQoL, viral loads and CD4) were unavailable for all participants lost to follow‐up at six months regardless of arm. Missing data were treated as missing at random. Potential covariates were not predictive of missingness, hence approaches such as propensity score adjustments were not feasible or even necessarily needed. We believe the missing at random assumption is reasonable with respect to no real patterns of missingness in the dataset as a function of potential predictors.

## 3 RESULTS

**Figure 1:**
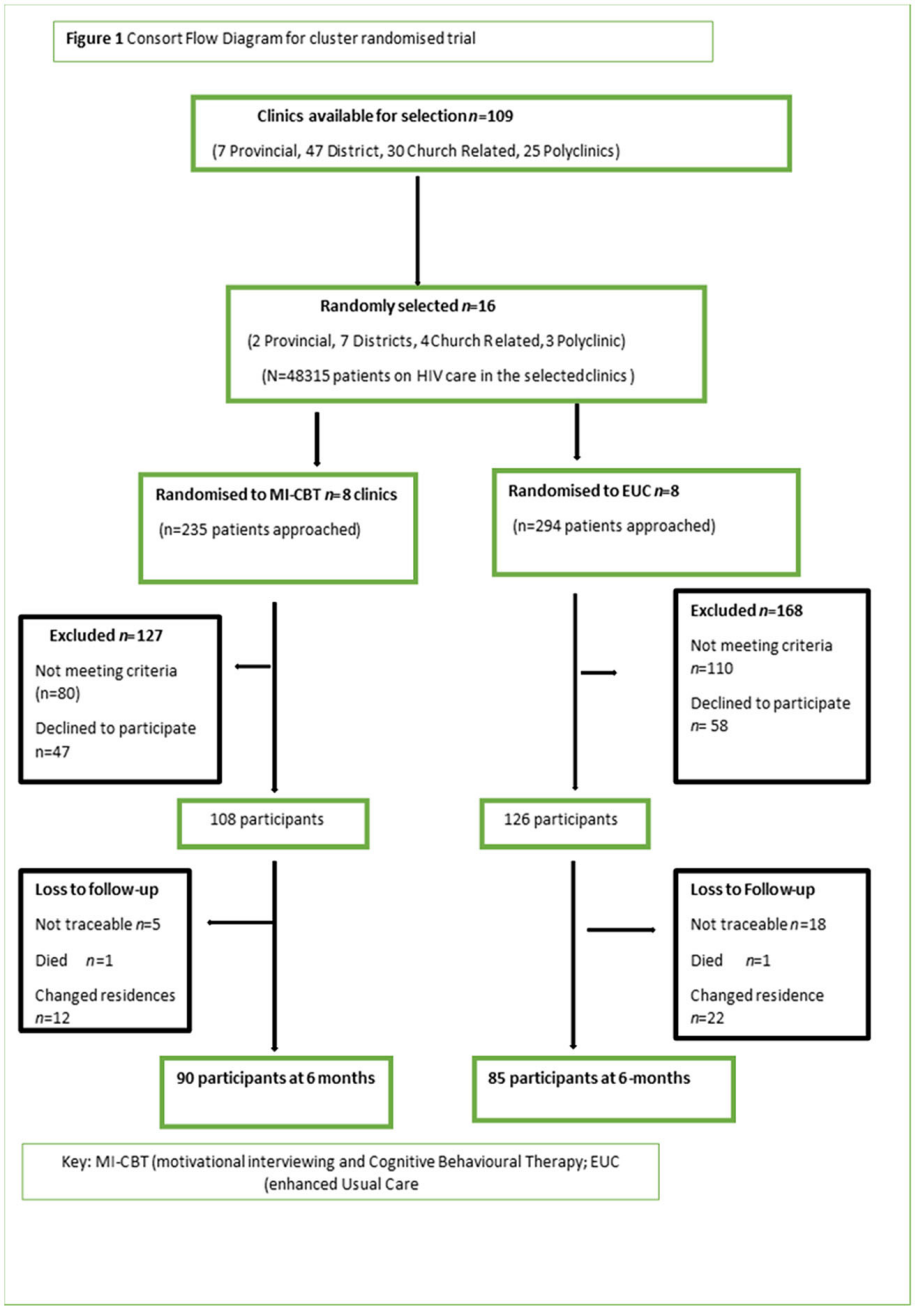
Consort flow diagram for cluster randomised trial.

Five hundred and twenty‐nine patients were eligible on the basis of their AUDIT score. However, 190 did not meet the other study criteria and 105 declined to participate. Two hundred and thirty‐four participants were recruited. Figure 1 is a consort diagram for the study. There were 108 (46%) participants in the MI‐CBT group and 126 (54%) participants in the EUC group. Participants were recruited from November 2016 to November 2017 and followed through to May 2018. Cluster sizes by clinic ranged from n = 5 to n = 21 with a median cluster size of n = 15. We retained 175 (75%) of participants at six months (83% in MI‐CBT and 67% in EUC) with no statistically significant difference in loss to follow‐up between the arms (*p* = 0.196). Two participants died of conditions related to HIV, and 57 (24%) could not be traced due to relocating from their place of residence. For the six‐month comparisons there was therefore roughly a 25% missing rate due to loss of follow‐up and across variables. Therefore, a total of 918 (85%) of sessions were delivered in the MI‐CBT arm and 351 (70%) delivered to the EUC arm. The number of sessions delivered to the two arms was not statistically significantly different between the arms (*p* = 0.49; Pearson correlation of 0.86).

Missing data at six months are primarily due to attrition. The planned attrition rate was 30%. The actual missing values were consistent across all measures and were 29% missingness due to attrition. There was not a specific variable that was differentially missing at a higher rate above 29%. Missing data due to causes other than attrition were negligible. Missingness due to causes other than attrition ranged from 0% to 3% across the set of six‐month endpoints. Differential missingness between the two treatment arms, was not significant.

Characteristics of the sample, broken down by trial arm, are shown in Table 1. More participants in the MI‐CBT were in paid work compared to those in the EUC (*p* = 0.03). The median number of sessions was 9 (range 4 to 10) in the MI‐CBT arm and 4 (range 3 to 4) in the EUC arm.

**Table 1:** Characteristics of participants in the two treatment arms, MI‐CBT and EUC

Variable sample (%)	MI‐CBT n = 108 [46]	EUC n = 126 [53]	*p*‐value
	M (SD)	M (SD)	
Mean age (SD)	43.6 (9.4)	43.0 (9.3)	0.15
Mean number of years in school (SD)	10.8 (3.2)	10.2 (2.9)	0.69
Gender (%)	n (%)	n (%)	
Female	17 (15.7)	33 (26.2)	0.11
Male	91 (84.3)	93 (73.8)	0.36
Marital status (%)			
Married	71 (65.7)	75 (59.5)	0.47
Divorced	11 (6.5)	12 (9.5)	0.23
Single	10 (9.3)	24 (19.1)	0.58
Widowed	7 (6.5)	8 (6.4)	0.21
Co‐habiting	9 (8.3)	7 (5.6)	0.63
Main work status (%)			
Paid work	38 (30.2)	27 (25.0)	0.03
Self‐employed	60 (47.6)	66 (61.1)	0.15
Non‐paid work	3 (2.4)	4 (3.7)	0.21
Student	3 (2.4)	5 (4.6)	0.11
Keeping house	4 (3.2)	3 (2.8)	0.27
Retired	2 (1.6)	1 (0.9)	0.02
Unemployed	16 (14.8)	2 (1.5)	0.07
Duration on antiretroviral therapy	5.36 (2.0)	5.12 (2.0)	0.72
ART appointment adherence in previous 3 months	87%	89%	0.56

EUC, Enhanced Usual Care.

### 3.1 Baseline assessments

At baseline, the mean AUDIT score for the MI‐CBT arm was 14.9 (SD 6.3) and for the EUC arm it was 14.7 (SD 6.2) (*p* = 0.81). The MI‐CBT arm had a higher log10 viral load 1.86 (SD 0.79) than the EUC arm 1.48 (SD 0.68) although this difference was not statistically significant (*p* = 0.13). Baseline CD4 count was similar between the arms with the MI‐CBT at 338.7 (SD 234.6) and the EUC at 338.7 (SD 234.6) (*p* = 0.37). There were no between‐group differences in mean WHODAS 2.0 or WHOQOL scores.

### 3.2 Fidelity

Use of intervention manual, recording of sessions both as audio‐tapes and notes, and use of participants’ intervention experience cards were the tools for maintaining intervention fidelity. Review of the 118 (10%) of sessions that were audio‐taped was compared with participants’ notes, as planned. The review of the audio‐tapes, intervention notes and participants’ experience showed fidelity to MI‐CBT at 86% and 79% for EUC.

### 3.3 Primary outcome

#### 3.3.1 AUDIT score

As shown in Table 2, at six months there was a statistically significant difference in AUDIT score between MI‐CBT and EUC arms, with a mean difference (95% CI) of −3.09 (−4.53; −0.01), *p* = 0.05). At Baseline, both participants in the MI‐CBT and EUC arm had mean AUDIT, scores of around 14.8. At six‐month post‐intervention, participants in the MI‐CBT arm had a mean score of 8.75, whereas participants in the EUC arm had mean scores of 11.61. Both arms had a statistically significant pre‐post reduction in AUDIT score at follow‐up compared to baseline: MI‐CBT mean difference (95% CI) −6.15 (−6.32; −6.00) *p* < 0.001 and EUC mean difference (95 % (CI) −3.09 (−3.21; −2.93) *p* < 0.001 arms.

**Table 2:** Primary and secondary outcomes in the active arm (MI‐CBT) compared to the EUC arm

	MI‐CBT	EUC
Primary outcomes		
AUDIT score	M (SD)	
Baseline AUDIT score	14.89 (6.31)	14.74 (6.22)
6‐month Mean Difference [95% CI]	−6.15 [−6.32; −6.00]***	−3.09 [−3.21; −2.93]***
Difference between MI‐CBT and EUC [95% CI]	−3.09 [−4.79; −1.23]*	
Secondary outcomes		
Viral load	M (SD)	
Baseline viral load log10	1.86 (0.79)	1.48 (0.68)
6‐month mean difference [95% CI]	−0.77 [−1.08; −0. 23]*	−0.40 [−1.02; −0.11]*
Difference between MI‐CBT and EUC [95% CI]	−0.37 [−0.45;0.12]	
Proportion with viral load undetectable	%	
Baseline proportion with viral load undetectable	28.72	37.71
6‐month mean difference [95% CI]	−0.77 [−1.08; −0. 23]*** *p* = 0.29	−0.40 [−1.02; −0.11]* *p* = 0.12
Mean difference between MI‐CBT and EUC [95% CI]	0.37	
CD4	M (SD)	
Baseline CD4 Count	338.7 (234.58)	442.6 (236.81)
6‐month mean difference [95% CI]	−29.93 [−96.3;36.5]	35.91 [−1.6;93.4]
Mean difference between MI‐CBT and EUC [95% CI]	−65.8 (−114.0;50.9)	
WHODAS	M (SD)	
Baseline WHODAS score	14.64 (2.98)	16.19 (4.57)
6‐month mean difference [95% CI]	0.95 [0.10;1.80]*	1.25 [0.39;2.52]**
Mean difference between MI‐CBT and EUC [95% CI]	0.3 [0.07;1.67]	
WHOQOL	M (SD)	
Baseline WHOQOL Score (SD)	86.33 (14.73)	84.43 (9.54)
6‐month mean difference [95% CI]	13.63 [10.11;17.24]***	12.21 [9.72;14.74]***
Mean difference between MI‐CBT and EUC [95% CI]	−1.22 [−4.85;2.41]	

AUDIT, Alcohol Use Disorders Identification Test; CI, Confidence Interval; EUC, enhanced usual care; MD, Mean Difference; SD, Standard Deviation; WHODAS, World Health Organization Disability Schedule; WHOQOL, World Health Organisation Quality of Life.

^*^ Effect size significant at the *p* ≤ 0.05 level

^**^ Effect size significant at the *p* ≤ 0.01 level

^***^ Effect size significant at the *p* ≤ 0.001 level.

### 3.4 Secondary outcomes

#### 3.4.1 Viral load

As shown in Table 2, there was a statistically significant reduction in viral load at six months, compared to baseline, or both with the MI‐CBT log mean difference (95% log (CI) −0.77 (−1.08; −0.15) *p* < 0.001) and the EUC log mean difference (95% (CI) log − 0.40 (−1.02; −0.11); *p* = 0.041) as shown in Table 2. However, the mean difference between MI‐CBT and EUC was not statistically significant (95% (CI) log − 0.37 (−0.45; 0.12; *p* = 0.46) at six months. The percentage of the detectable viral load at baseline was 28.70% (>40 copies per mL). The drop we reported in viral load is very encouraging and is consistent with a meta‐analysis of studies of interventions aiming to improve adherence in HIV which showed that participants who received an intervention were 1.25 times as likely to achieve an undetectable VL as participants in a control arm. At baseline slightly more in the MI‐CBT arm than in the EUC arm were suppressed (37.7% vs. 28.7%). At follow‐up, 89% in the MI‐CBT arm and 86.4% had virological suppression.

#### 3.4.2 CD4

At six months there was no statistically significant change in CD4 count for either arm and no difference between arms: MI‐CBT mean difference (95% (CI) −29.88 (−96.28; 36.50) *p* = 0.38); EUC mean difference (95% (CI) 35.92 (−21.57; 93.41) *p* = 0.22); Effect size mean difference (95% (CI) −31. 55 (−114.03; 50.93) *p* = 0.45.

#### 3.4.3 Functionality

There was a statistically significant improvement in functionality for both interventions: mean difference (95% (CI) 0.95 (0.10; 1.80); *p* = 0.029 and EUC mean difference (95% (CI) 1.25 (0.39; 2.52) *p* = 0.01 arms, but group differences were not significant: mean difference (95% (CI) 0.3 (0.07; 1.67) *p* = 0.67.

#### 3.4.4 Quality of life

Quality of life improved significantly in both arms between three and six months: MI‐CBT MD (95 % CI) −13.63 (−17.05; −10.21), *p* < 0.001; EUC MD (95 % (CI) −12.21 (−14.71; −9.72) *p* < 0.001. However, there were no statistically significant between‐group differences MD 95% (CI) −1.22 (−4.85; 2.41); *p* = 0.51, as shown in Table 2.

## 4 DISCUSSION

This is one of very few randomized clinical trials in a low‐income African country in people living with HIV and co‐existing alcohol use disorders to show that interventions task‐shared to general nurses led to declines in alcohol use, general function and viral load. This was the case for participants who received an average of nine‐sessions of a psychological intervention based on motivational interviewing with brief CBT, and also for those who received a brief package of 3 to 4 sessions of psychoeducation with personalized feedback on alcohol use. Trial results also show that the MI‐CBT intervention was superior in reducing alcohol use, based on the AUDIT score, at 6‐month follow‐up. At baseline, both participants in the MI‐CBT and EUC arm had mean AUDIT scores of around 14.8. This was within the upper band of harmful drinking. According to cut‐off scores described by Nadkarni and colleagues, the mean score of 8.75 at six‐month post‐intervention for the MI‐CBT arm still indicates harmful drinking, however at a lower end of the severity band, whereas the mean score of 11.61 in the EUC arm indicates harmful drinking, closer to the upper end of the band [39]. Based on validation of the AUDIT scale in multiple countries, the decrease in AUDIT score we demonstrated in the MI‐CBT arm suggests a drop from a highly hazardous or even harmful level of alcohol intake with a high risk of dependence to a level which has a low to moderate risk of hazard and a low risk of dependence [32]. However, in the absence of validated AUDIT cut‐points for Zimbabwe we can only make this assertion tentatively.

Our findings are in keeping with a systematic review from 2017 assessing the effect of behavioural interventions on alcohol use in PLWH, which found a reduction in drinking behaviour [46]. Our findings strengthen the preliminary evidence from a small RCT in Kenya among HIV‐outpatients, consisting of a culturally adapted group CBT, which found a statistically significant difference in self‐reported alcohol abstinence at 90‐day follow‐up [13], and from a trial of a four‐session intervention in women living with HIV in South Africa who had heavy drinking [10]. In a follow‐up study, Papas showed CBT‐based intervention to reduce mean drinks per day and percentage drinking days thus supporting the use of psychological interventions in alcohol use. The drop in viral load, and the increase in those with viral suppression, was not statistically significant between MI‐CBT and the EUC arms but improved from baseline in each arm suggesting a clinically important effect of these interventions on adherence. We may have been able to demonstrate a stronger effect of the intervention on reducing viral load if we had targeted this intervention at participants with baseline viral non‐suppression. By including those with viral suppression we may have diluted the effects on viral load. This RCT indicates that task‐sharing can be an effective approach for addressing AUD in PLWH. Nurses in Zimbabwe are respected, educated and well‐trained. Across SSA where trained nurses provide HIV care, there are opportunities to capacitate these nurses to manage mental health comorbidities, such as problematic alcohol use [21, 47]. Evidence‐based interventions, such as MI‐CBT for AUD, can be effectively delivered by up‐skilling existing staff [20].

One reason for our findings could be that we used many sessions, both in the active and the EUC arms. We were influenced by evidence from Project MATCH in terms of content of the intervention and adapted this to a format that the nurses could provide in the context within their usual clinical sessions [29]. Another reason could be that we used personalized feedback in both the MI‐CBT arm and the EUC arm. Personalized feedback is described in more detail earlier but essentially comprises telling the person their AUDIT score and explaining what this score means in terms of risk of harm to body organs and to risk of social harms. We were influenced in choosing our intervention by a prior study in South Africa which found that a three‐session motivational‐skills building risk‐reduction intervention in PLWH was associated with a reduction in the use of alcohol [48]. We think the number of sessions in our MI‐CBT was reasonable given the complexity of co‐morbid AUD and HIV. Future research needs to look at options for providing session material in groups [10, 49], although group therapy can be challenging to arrange in routine clinical settings in LMIC given distances from clinics and transport costs. Delivery via mobile technology could be another option given the wide use of phones in SSA, and technology has been shown to be a useful vehicle for MI‐based interventions in the US [9, 50].

This cluster RCT has several limitations. First, cluster RCTs have diminishing returns in precision and power as the size of the cluster increases. In order to mitigate this in our trial, we determined the number of clusters and cluster size concurrently. To try to limit selection bias, which is another concern with cluster RCTs, facilities were selected through stratified randomization and participants were randomly selected within clusters. Individuals less easy to be contacted are likely to be under‐represented as the study funds did not allow for researchers to visit everyone who could not be contacted by phone or through normal clinic outreach. Second, participants were not and could not be blinded to the allocation which was a limitation of our study. However, the outcome assessors were blinded to the arm of the facility. Third, our study had differential loss to follow‐up (which was, however, not statistically significant) with more participants lost in the control arm, which is a limitation. The loss to follow‐up was greater in the EUC arm than in the MI‐CBT arm. The effect of the MI‐CBT intervention on reduction in alcohol use and in viral load was greater than the effect of the EUC intervention, although this was only significant at the 0.05 level for alcohol use and was non‐significant in the case of viral load. If the follow‐up had been more equal, we might have been able to demonstrate a stronger effect of the intervention on alcohol use and even on viral load. Given that the loss to follow‐up was greater in the EUC arm it is possible that the MI‐CBT intervention was more effective than EUC in promoting better engagement in care. Fourth, a limitation of our study is that alcohol use was measured with the AUDIT which is a self‐report questionnaire and subject to social desirability bias [51]. Social desirability may have led to bias in the reporting of reduced alcohol use in the intervention arm. We think this is unlikely to fully explain differences between the two arms given that variables that are not reliant on self‐report, such as viral load, also improved more in the intervention arm. Self‐report methods are considered a reliable and valid approach to measuring alcohol consumption [52]. While future research should include biomarkers such as phosphatidyl ethanol (PEth) it is worth noting that in the absence of a gold standard biological measure for alcohol it is difficult to explain discrepancies between self‐report and PEth that have been documented in other studies [53, 54]. For the 6‐month comparison, there was roughly a 25% missing rate due to loss of follow‐up and across variables. This is a limitation of the study but, as the dropouts were random, it implies a loss of statistical power but still valid results. The AUDIT, WHODAS and WHOQOL have not been validated in PLWH in Zimbabwe, although they have been validated in other countries in SSA, and Zimbabwe was one of the study sites in their development [32, 44, 55]. In the absence of a validated AUDIT cut‐off in this population, we used cut‐offs from other studies. We acknowledge that we used a lower‐cut‐point than previously used levels in the general population. The WHO guidance [31] on the use of the AUDIT emphasizes that selection of the cut‐off point should be influenced by national and cultural standards. Data from validation of the AUDIT advise “Scores of 6 to 7 may indicate potential harm for groups more susceptible to the effects of alcohol, such as young people, women, the elderly, people with mental health problems and people on medication” [31]. We used a lower‐cut‐point because of evidence that PLWH who drink the same amount as HIV‐negative people have higher blood alcohol levels and a greater risk of liver damage; because locally brewed alcohol drinks in common use in Zimbabwe tend to have high levels of ethanol; because of lack of validation of precise AUDIT cut‐points in Zimbabwe; and because alcohol consumption per capita is less across low‐income African countries than in the US and Europe, especially among women [56, 57]. Therefore, we chose a conservative threshold given our population under investigation and described drinking patterns in Zimbabwe. Research is needed to validate cut‐points for AUDIT in Zimbabwe and other low‐resource African countries as it remains unclear as to what the decrease of the AUDIT score by 3 units means clinically and is thus another limitation of the conclusions we can draw from this study. A further limitation was that the cluster size was not equal across all sites. The intention was to recruit 15 eligible participants per cluster, however, due to time and resource constraints, including minimal study funding, in some cases recruiters had to move onto the next facility without having recruited 15 participants, ending up with a range of 6 to 21 per site. Another limitation is that potential participants less easily to be contacted are likely to be under‐represented in those recruited. This study was carried out for a PhD for the lead author (MM) and thus explains the time constraints. Further funding and time would have allowed recruiters to stay for longer at each site, and for researchers to visit to screen everyone from the random sample of potential participants. Another limitation is that due to lack of funds, follow‐up was limited to six months. Examining longer term maintenance effects of AUD interventions should be built into the design of future trials.

This has important implications for practice and policy, and for Sustainable Development Goals, suggesting that personalized feedback on alcohol use could be incorporated into regular HIV follow‐up for those with hazardous drinking. The MI‐CBT intervention showed that effects are maintained at six months although the authors would want to understand the effects of the intervention over a long period of time.

## 5 CONCLUSIONS

This cluster RCT showed that an MI‐CBT intervention can be effectively implemented by non‐specialist providers and led to improvement in a number of clinical outcomes. Monetary incentives may be given to the registered general nurses to ease their burden of performing extra duties due to an increased work load. A next step is the evaluation of the cost‐effectiveness of these interventions that takes the complexity of current HIV treatment settings and current staffing into account, and which actively involves people with lived experience of HIV and alcohol use in the design of these trials.

## COMPETING INTERESTS

The authors declare that they have no competing interests.

## AUTHORS’ CONTRIBUTIONS

MM and SS conceived the study and developed the concept. MM, SS and MA developed the protocol of the study. AC, DC and SR critically reviewed the protocol and MK and AH developed the data analysis plan and carried out the data analysis. MM, SS, MA BD, MD, WM and TM developed the interventions. GDM and DTZ participated in the development of the manuscript. MM and MA wrote the first and second drafts together. All authors reviewed the manuscript and approved its submission.

## ABBREVIATIONS

ACASI, Audio Computer Assisted Self‐Interview; AUD, Alcohol Use Disorders; AUDIT, Alcohol Use Disorders Identification Test; EUC, Enhanced Usual Care; LMIC, Low‐and Medium‐Income Countries; MI‐CBT, Motivational Interviewing –Cognitive Behavioural Therapy; PLWH, People living with HIV; RCT, Randomized Controlled Trial; SSA, Sub‐Saharan Africa; WHO mhGAP IG, World Health Organization mental health gap intervention guide?; WHODAS, World Health Organisation Disability Assessment Schedule; WHOQOL, World Health Organisation Quality of Life.
